# Account of Diseases in an Eastern District of London, from the 20th of March to the 20th of April, 1800

**Published:** 1800-05

**Authors:** 


					State of Difeafes in London. 407
STATE OF DISEASES IN LONDON.
Account of Difeafes in an Eajiern DiJiriSl of London, from the 20ih of
March to the loth of April, I boo.
JNo. oi Cales.
ACUTE D.ISEASES.
Typhus gravior - - z
Typhus mitior - - 3
Peripneumonia notha - 4
Acute Rheumatifm - 3
CHRONIC DISEASES.
Cough - - - 13
Dyfpnoea - 9
Cough with Dyfpnoea - 15
Haemoptyfis 3
Pleurodyne - - -2
Phthifis Puftnonalis - 4
Galtrodynia - - 8
Dyfpepfia - -7
Diarrhoea - - - 12
Bilious Vomiting - 3
Enterodynia - 4
Obftipatip 2
Hasmorrhois - - - 5
Nephralgia Calculofa - 2
No. of Cafes.
Hydrothorax - - 1 3
Anafarca - - -.5
Afcites 4
Cephaloea ? - 7
Apoplexy 2
Vertigo - - 3
Hyfteria - - - z
Ophthalmia ? - 3
Chronic Rheumatifm - 20
PUERPERAL* DISEASES.
Peritonitis 3
Menorrhagia lochialis - 5
Dolores poft partum - 3
Maftrodynia - 2
INFANTILE DISEASES.
Ophthalmia - 3
Ophthalmia purulenta - 2
Convulfio - - -.'2.
Vomitus - I
The confiderable change which has taken place in the ft ate
of the weather, during the laft few weeks, has produced, as
plight be expected, an alteration in the fhte of difeafes,?
G g g 2 The
The number of pneumonic complaints, which have lately
formed fo large a proportion of the lift, is fmaller; though fe-
veral patients, included in a former lift, are ftill labouring un-
der the remaining effe&s of thofe difeafes. %-
Since the change of the weather, there have occurred a few
cafes of fever; but thefe have been 'attended with fo mild a
train of fymptoms, as not to occafion any confiderable appre-
henfion.
Diforders of the ftomach and bowdls ftill continue trouble-
fome, but are likely to be removed by a gentle diarrhoea,
which pretty generally prevails.
The great mortality which has occurred during the winter,
has been the fubje?t of very general remark. It appears from
the lift fubjoined to this report, which has been taken from the
weekly bills of mortality, that the number of deaths of aged
perlbns, and from thofe difeafes which are moft prevalent in
the winter months,- has been very confiderable. From a coifl-
parifon with fome former lifts, the proportion feems to have
j been nearly as two to one. ,
The Deaths in the Bills of Mortality for the lafi 'Three Months are
, i ?, Jiated as follow:
Aged
Apoplexy '
Allhma
Cancer - -
Child-bed
Confumption
Convulfions
Dropfy
Fever
Gout
Hooping Cough
Inflammation ~
Lunatic - *? - 39
Meafles - - 140
Mortification 74
Pal fy - - 37
Pleurify - - 13
Small-Pox - - I7f
Still Born - ^ - 113
Suddenly . - - 44
Teeth - - - iiq
Thrufh - - 6
Water in the head - 11
Worms 7

				

